# Medication Adherence in a Community Population with Uncontrolled Asthma

**DOI:** 10.3390/pharmacy8040183

**Published:** 2020-10-07

**Authors:** Sarah Serhal, Bandana Saini, Sinthia Bosnic-Anticevich, Ines Krass, Frances Wilson, Carol Armour

**Affiliations:** 1 Woolcock Institute of Medical Research, 431 Glebe Point Road, Glebe, NSW 2031, Australia; bandana.saini@sydney.edu.au (B.S.); sinthia.bosnic-anticevich@sydney.edu.au (S.B.-A.); f.wilson@sydney.edu.au (F.W.); carol.armour@sydney.edu.au (C.A.); 2 School of Pharmacy, The University of Sydney, A15, Science Rd, Camperdown, NSW 2006, Australia; ines.krass@sydney.edu.au; 3 School of Medical Sciences, Faculty of Medicine and Health, University of Sydney, Camperdown, NSW 2006, Australia

**Keywords:** asthma, allergic rhinitis, medication management, pharmacy, primary care, adherence

## Abstract

It is well documented that the use of medications in asthma and allergic rhinitis is often suboptimal, and consequently, patients remain symptomatic. This study aimed to determine the extent and type of medication-related issues contributing to poor asthma control by profiling medication management in those most at risk—a population with clinically uncontrolled asthma. Participants (*n* = 363) were recruited from Australian community pharmacies, and a dispensed medication history report for the previous 12 months was collected to examine medication adherence and factors affecting adherence. Information was also collected regarding participant asthma control and asthma/allergic rhinitis (if applicable) management. The participants’ mean asthma control score was 2.49 (± 0.89 SD, IQR = 1.20) (score ≥ 1.5 indicative of poorly controlled asthma), and 72% were either non-adherent or yet to initiate preventer therapy. Almost half had been prescribed high doses of inhaled corticosteroid and 24% reported use of oral corticosteroids. Only 22% of participants with concomitant allergic rhinitis were using first line treatment. A logistic regression model highlighted that participant health care concession status and hospital admissions were associated with better adherence. Suboptimal medication management is evident in this at-risk population.

## 1. Introduction

Despite the availability of effective medications, asthma is responsible for 1000 deaths each day globally, and is amongst the top 20 causes of years of life lived with disability [1]. Optimal therapeutic management of asthma is needed to achieve better health-related quality of life, reduce patient and societal burden, and significantly improve patient clinical outcomes.

Asthma often occurs with a range of comorbid conditions, of which allergic rhinitis is the most common, given the shared inflammatory pathophysiology and physio-anatomic continuity between the upper and lower respiratory tracts [2,3,4,5,6,7,8,9,10,11,12]. Epidemiological data indicate that about 80% of people with asthma have allergic rhinitis [13]. Poor allergic rhinitis control is therefore a significant risk factor for poor asthma control [2,3,7,8].

With the right medication, at the right dose, used with the correct technique, asthma and allergic rhinitis can be well controlled, and to achieve long-term control ongoing medication use is often required. However, the quality use of asthma and allergic rhinitis medications may be compromised at various levels of the patient care chain, and has been reported internationally [14,15,16,17,18]. Poor levels of adherence have been observed globally, reasons for which have been the focus of much research. A plethora of medication, patient and external factors drive both unintentional and intentional poor adherence [18,19,20,21,22,23,24,25,26]. Drivers often vary internationally, dependent on the sociopolitical context in which they are explored [20].

International standards for asthma management are prescribed by the Global Initiative for Asthma (GINA), and interpreted locally in Australia by the National Asthma Council Australia (NAC) [13,23]. It is recommended that all adults with asthma be treated with a preventer (inhaled corticosteroids (ICS)) to control symptoms, and to reduce the risk of future exacerbations and decline in lung function by reducing airway inflammation [13,23]. Reliever medications (short-acting beta 2 agonists (SABA)) are used by patients on an as-needed basis to relieve worsening symptoms or exacerbations [13]. Recently, GINA Guidelines have recommended the use of corticosteroid-containing inhalers for acute symptoms. The current guidelines recommend matching treatment to asthma control and risk of exacerbations in a stepped approach with frequent patient review, to ensure minimum long-term exposure to high-dose preventers [13]. Prescribers can scale down or step up therapy based on patient responses [13]. Most adults with asthma should be controlled on regular low-dose ICS preventer (Step 2) [13]. The goal and ultimate measure of success of asthma management is to eliminate the frequent need for emergency use of reliever medications and oral corticosteroids [13,23].

In Australian primary care, prescribing patterns that are not guideline concordant have been observed, for example the level of combination preventer (ICS and long-acting beta2-agonist (LABA)) prescribing is much higher than expected based on asthma control reported by patients [27]. Even when prescribed optimal levels of therapy, many adults with asthma do not adhere to the daily use of preventers, relying instead on relievers [14,16]. Data from the Australian Centre for Asthma Monitoring [27] showed that a third of the patients prescribed preventer inhalers (ICS) had it dispensed only once in 12 months, which differs from the guideline-based objective of adults being on regular low-dose inhaled corticosteroid to limit airway remodeling and maintaining control [27]. Even when taking preventers (ICS), many patients cannot correctly use inhalers as recommended, leading to sub-optimal dosing and unnecessary side effects [28,29,30,31]. Further, the rising costs of therapeutic management have been shown to play a role in patient decision-making regarding adherence to treatment [21,32]. Both direct and indirect expenses associated with the purchase of prescription preventer medications (ICS) for asthma can deter patient adherence and lead to a preference for relying on the less expensive reliever medications that can be purchased without a prescription in Australian pharmacies [21]. 

Topical anti-inflammatory and oral antihistamines for allergic rhinitis treatment can also be purchased directly from Australian pharmacies, and consequently people with allergic rhinitis often self-select medications in the pharmacy without consulting a health care professional [17], and make their decisions either experimentally or based on their own experience [33]. A recent Australian survey indicated that only 15% of surveyed allergic rhinitis patients presenting at pharmacies left with an appropriate medication [17]; most with oral antihistamines and few with the more appropriate intranasal corticosteroids. Whilst oral antihistamines may be a short-term preference in patients with asthma, given that they are cheaper and easier to use, in the long term, this may translate to poorly controlled asthma, necessitating frequent use of preventer asthma medications at higher doses. This consequence is a result of post nasal drip and upper airway irritation, which augment lower airway symptoms [34,35]. Therefore, the treatment of allergic rhinitis is vital for the effective management of asthma, and intranasal corticosteroids are the first-line treatment for people with allergic rhinitis and coexisting asthma [36].

Suboptimal therapeutic management of asthma and allergic rhinitis has previously been studied separately. This study aimed to determine the extent and type of medication-related issues contributing to poor asthma and allergic rhinitis control by profiling medication management in a single population most at risk—a population with clinically uncontrolled asthma.

## 2. Materials and Methods

The study involved a cross-sectional observational study of patients presenting at their local community pharmacy between August 2018 and February 2019. All participants provided informed consent prior to enrolling in the study.

This research was part of an implementation trial approved by the Human Research Ethics Committees of The University of Sydney, Curtin University and The University of Tasmania, and funded by the Australian Government Department of Health via the 6th Community Pharmacy Agreement [37]. The implementation trial was a two-arm clustered randomized controlled trial that aimed to assess the impact of a specialized pharmacy-based intervention on asthma control compared to standard (control) care [37]. This paper analyzed baseline medication-use data collected from recruited participants with asthma in the above trial.

### 2.1. Pharmacy Recruitment 

Pharmacists from regional and metropolitan areas in New South Wales, Western Australia, and Tasmania, were invited to self-nominate their interest in participating in the study via an online expression of interest form sent out by the Pharmacy Guild of Australia. Pharmacies were stratified by geographical distribution to be representative of the general population, and selected to participate using random number generation. Geographic remoteness was determined as per the Pharmacy Accessibility Remoteness Index of Australia (PhARIA)—(high accessible (PhARIA 1), accessible/moderately accessible, remote and very remote (PhARIA 2–6)) [38].

### 2.2. Participant Recruitment

Pharmacies were asked to recruit a minimum of 7 asthma participants each. The classification of asthma was based on patient self-report. The sample size was based on feasibility established in previous studies to account for predicted pharmacy and participant dropout rates and the numbers required to show significant change in the larger implementation trial [37,39,40].

#### 2.2.1. Participant Inclusion Criteria

The primary inclusion criteria for participants included uncontrolled asthma as determined by a score ≥ 1.5 in the Asthma Control Questionnaire (ACQ) [41,42]. Patients aged ≥ 18 years, who (1) were able to communicate with the pharmacist in English, (2) were a regular patient of the pharmacy (receiving medications from that pharmacy for the previous 12 months and having a dispensing history available) and (3) managed their own medications, i.e., patients were not dependent on carers (as determined by the pharmacist), were included if they consented to participate. 

#### 2.2.2. Participant Exclusion Criteria

Participants were excluded from the study if they (1) had a high dependence on medical care (more than 5 morbidities and specialist care), (2) were unable to manage their own medications (as determined by the pharmacist) (3) had a confirmed diagnosis of chronic obstructive pulmonary disorder (COPD) (self-reported by the participant) or (4) had a terminal illness.

### 2.3. Data Collection

Participants took part in a face-to-face comprehensive assessment of current asthma management. A web-based clinical decision support program linked to pharmacy dispensing software guided all data collection and intervention delivery. All participant responses were de-identified prior to the research team receiving the data. 

#### 2.3.1. Asthma Control

Asthma symptom control was assessed via the ACQ [41]. 

#### 2.3.2. Asthma History 

Self-reported age of asthma symptom onset, smoking status and whether a participant had received a lung function test in the last 12 months were the data collected, along with demographic information including age, gender-identity and location of residence (urbanity/rurality range).

#### 2.3.3. Health Care Utilization

The self-reported number of hospitalizations and accident and emergency visits in the last 12 months was recorded.

#### 2.3.4. Medication History and Adherence 

Asthma medication profiles were generated for each participant using dispensed medication history data for the previous 12 months, automatically extracted from the pharmacy dispensing software. These dispensed medication reports were used to determine participant adherence to asthma medications over the 12-month period by calculating the Proportion of Days Covered (PDC), which refers to the proportion of days covered by medication dispensed (Equation (1)) [43,44,45].

(1) $$PDC=\left(\frac{Number\;of\;days\;in\;the\;period\;“covered”}{Number\;of\;days\;in\;period}\right)\;\times \;100\%$$

Equation (1) Proportion of Days Covered formula. *Number of Days in the period “covered”* refers to the number of days the participant was covered by at least one asthma preventer medication based on the dates a prescription was dispensed, the number of devices per script, the actuations per device and the participant’s prescribed dose. *Number of days in period* refers to the number of days between the dates of first supply of an asthma preventer medication and the date of data collection.

The PDC was only calculated for participants who had a minimum of three preventers dispensed on separate occasions in the preceding 12-month period. For the purpose of analysis, adherence was dichotomized to a PDC of 80% or greater (adherent) or a PDC of less than 80% (non-adherent) [46]. In case no instructions for medication administration appeared in the medication record or in cases where dose variability occurred, standard dosage was used to calculate the number of days covered for each medication dispensed. 

Differences in characteristics between participants classified as adherent or non-adherent were explored. Further, information on asthma-related drug classes, individual medications and inhaler device type being used by participants was collated and used to compare against guideline recommendations and to determine if medication and formulations affect adherence. 

#### 2.3.5. Current Medication Management

Based on dispensed medication history data for the previous 12 months, medications were classified as *current* if they were dispensed within the 3 months prior to data collection. Using these data, we explored the current medication management of participants.

#### 2.3.6. Allergic Rhinitis

All participants were asked if they had a diagnosis or were experiencing symptoms of allergic rhinitis. A proportion of participants (in accordance with the larger implementation trial [37]) were asked if they were treating their allergic rhinitis symptoms, and if so, to specify medications being used.

### 2.4. Data Analysis

Cross-sectional data collected by the project specific software were exported as an Excel spreadsheet and then imported into SPSS Version 25, where descriptive statistics were applied. 

To explore predictors of adherence, categorical variables were compared using Pearson’s Chi Square test, and continuous variables were explored using a Mann–Whitney U test. A significance level of *p* < 0.05 was used for all statistical procedures. 

A forward logistic regression was performed using variables shown to be significantly associated with adherence following initial exploratory analysis. To test the goodness of fit of the model, the Hosmer and Lemeshow test was used.

## 3. Results

### 3.1. Participant Characteristics

A total of 363 eligible participants was recruited into the study by 95 community pharmacies. Participant characteristics are detailed in Table 1.

### 3.2. Asthma Control

Participants reported a mean ACQ score of 2.49 (±0.89 SD), scores ranged from 1.50 to 5.67.

### 3.3. Asthma Medication Management Over Preceding 12 Months

Of the 363 participants, only 80% had at least one asthma ICS preventer dispensed in the preceding 12 months, with combined ICS + LABA (76%) being the most dispensed drug class (Table 2). Seretide (fluticasone propionate/salmeterol xinafoate) pMDI 250 mcg/25 mcg was the most dispensed preventer medication. A full list of the respiratory medications dispensed in the preceding 12 months by formulation, trade name and strength is presented in Appendix A. 

Over the 12-month period, the total number of different asthma preventer medications taken by participants ranged between 1 and 3, medicines with a mean of 1.2 (±0.6).

Pressurized metered dose inhalers (pMDI) were the most common device type dispensed over the 12-month period (43%) (Table 3), with 74% of participants collecting at least one pMDI device, whether for preventative or reliever therapy. Add-on therapy was dispensed for 24% of the population, with the most common drug class being a long-acting muscarinic antagonist (LAMA). Almost one-quarter (24%) of participants had oral corticosteroids dispensed in the previous 12 months. 

### 3.4. Adherence to Preventer Therapy

**Figure 1:**
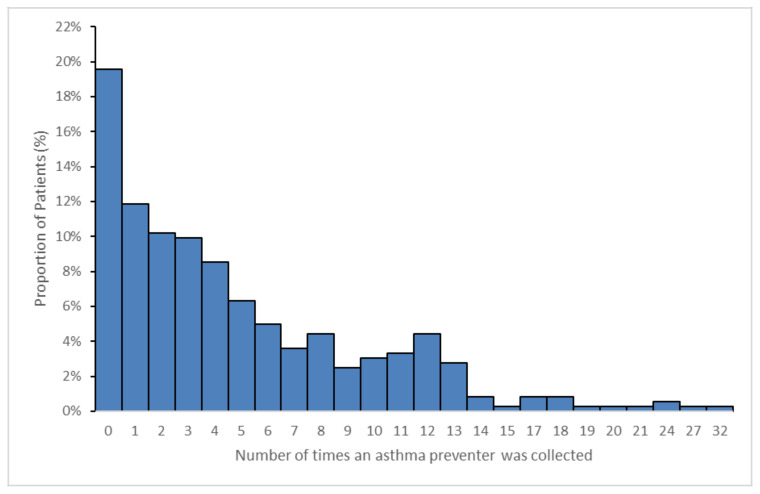
Number of times an asthma preventer medication was collected by participants over a 12-month period (*n* = 363).

Participants collected an asthma preventative medication, on average, five (±5.1 SD) times over the 12-month time period. This ranged from 0 to 32 times over 12 months, as presented in Figure 1.

Of the 363 participants, 42% (*n* = 151) had preventative medication dispensed less than three times, and therefore a valid PDC score could not be calculated. These participants were deemed non-adherent for the purposes of this study. 

For the remaining 212 participants, the PDC was calculated. Only 49% (*n* = 103) of these participants were reported as adherent (PDC ≥ 80%). 

Thus, out of all the participants (*n* = 363), only 28% (*n* = 103) were considered adherent to their preventative therapy, with 52% (*n* = 189) non-adherent to preventer therapy and 20% (*n* = 71) having no preventer dispensed at all in the preceding 12 months. 

### 3.5. Factors Associated with Adherence

Univariate analysis showed statistically significant positive associations between the number of hospital presentations (*p* = 0.009), older participant age (*p* = 0.026), confirmed health care concession status (*p* = 0.001), sole use of an Ellipta device (*p* = 0.042), use of a LABA only medication (*p* = 0.038), and use of a LAMA + LABA combination medication (*p* = 0.046) with a participant being adherent. There was a negative association between sole use of a Turbuhaler (0.004) and adherence.

There were no significant relationships between adherence and participant location (state or remoteness), gender, age of asthma onset, lung function status, smoking status, presence of comorbid allergic rhinitis, hospital admissions, exacerbations (indicated by oral corticosteroid in participant medication history), other device types or medication class, number of different asthma preventer medications and devices used over the previous 12 months, or medication dosage or device type variability that occurred in the previous 12 months. These variables were not included in the final model.

A forward logistic regression consistently selected or retained greater than one hospital presentation (OR = 8.386 (95% CI: 2.049, 34.326)) and health care concession status (OR = 0.365 (95% CI: 0.196, 0.680)) as variables associated with better adherence, and sole use of a Turbuhaler preventer (OR = 3.077 (95% CI: 1.322, 7.160)) was associated with poor adherence. This model fitted the data well (Hosmer and Lemeshow test χ^2^ = 2.92, df = 4, *p* = 0.570).

### 3.6. Current Medication Management

In the 3 months prior to data collection, as an indicator of current asthma management, preventer therapy was present in the records of 86% (*n* = 240) of these participants. ICS/LABA combination was the most used drug classes (80%). Nearly half (45% *n* = 108) of these participants had been dispensed high-dose ICS or ICS + LABA. Very few were on low-dose ICS (Table 4). 

The total number of asthma preventer medications taken currently by participants ranged from 1 to 3 medicines, with a mean of 0.67 (±0.60). 

The combinations of respiratory medications taken by these participants are presented in Appendix B. Of these participants, 9% were using SABA alone to manage their asthma. The most prevalent combination of therapy was ICS + LABA and a SABA, used by 31% of participants. Add-on therapy was used by 25% of participants.

### 3.7. Allergic Rhinitis 

Allergic rhinitis was reported by 71% (*n* = 259) of participants.

#### Allergic Rhinitis Management

A subset of participants (*n* = 152) who reported symptoms of allergic rhinitis were asked if they were treating their allergic rhinitis, the results of which are depicted in Table 5. Of these participants, 49% were using medications to help manage allergic rhinitis symptoms. Sole therapy with an oral antihistamine was the most common management strategy (20%). Where a participant was taking more than one medication, the most common combination was oral antihistamine and intranasal corticosteroid (9%). Only 22% of the total 152 participants with poorly controlled asthma who were asked about their allergic rhinitis management used an intranasal corticosteroid.

## 4. Discussion

Our study was able to profile asthma and allergic rhinitis within a single at-risk population. Based on the findings, a significant gap remains between evidence-based guidelines and current medication management in people with asthma. It is known that the appropriate use of medications can significantly improve therapeutic outcomes for people with asthma; thus, monitoring pharmacy medication dispensing records provides a mechanism for recognizing the under-use of medications and identifying the concordance of asthma management with evidence-based guidelines [27]. By profiling medication management in those most at risk—a population with clinically uncontrolled asthma—this study found that medication adherence and suboptimal treatment were significant issues. 

All the participants in our study had poorly controlled asthma as assessed by the asthma control questionnaire (ACQ) [41]. This is a recognized method for assessing the risk of future exacerbations and identifying symptom severity [41,42,47,48]. Guidelines state that most people with asthma should be well controlled on a low-dose ICS alone—Step 2 therapy [13]. In the previous 12 months, 42% of our participants (*n* = 151) had preventer medication dispensed fewer than 3 times, whereas for optimal control they should have had 11–12 prescriptions dispensed. When we explored the proportion of days covered by preventer medication, under half of those for which a value could be calculated were adherent. Thus, it is not surprising that their asthma was not controlled.

A previous cross-sectional study which surveyed adults with asthma (*n* = 2686) in an Australian context found that 57% of the population that reported uncontrolled asthma symptoms were non-adherent or were not using a preventer. Our study found that 72% of participants with uncontrolled asthma symptoms appeared to be non-adherent or were not using a preventer. This indicates that suboptimal adherence amongst poorly controlled asthma participants is a larger problem than earlier estimates suggest, and remains unresolved. 

When reviewing the strength of ICS being used by study participants, 93% had been prescribed medium (48%) to high (45%) ICS doses. Very few were on low-dose ICS. These data support previous evidence that higher doses of ICS may be overprescribed in Australia [49]. Additionally, one in four participants had used oral corticosteroids in the previous 12 months—indicating a lack of asthma control. These data suggest that there may be problems regarding the prescribing of asthma medications, and that undetected suboptimal adherence may be interpreted as poor therapeutic response, perpetuating a cycle of uncontrolled asthma symptoms, review and therapy escalation. The results may also suggest the presence of severe or difficult-to-treat asthma [23]. The high prevalence of poor adherence to preventer therapy or a lack of preventer therapy is consistent with international studies, despite variations in thresholds and measurements used to classify adherence [50]. For example, a study conducted in the United States of America (USA), using the PDC method and a cut off of 80% or greater for adherence, found that only 20% of fluticasone propionate users (an ICS) were adherent to therapy over a one-year period [50,51]. A European study exploring asthma control and management in 8000 patients found only 48% self-reported using their preventer everyday [52]. A study from the United Kingdom found that up to 76% of patients with asthma used fewer than 10 ICS canisters in a year, which was based on the number of cannisters prescribed [50,53]. There is a clear need for standardized measures of adherence for respiratory measurement to allow for global comparisons in asthma maintenance.

All participants within this study cohort should be on preventer therapy. The 20% of participants who were not on preventive therapy represent people with asthma who are falling between the cracks of primary care. As we can only report on what a participant had chosen to get dispensed at the pharmacy, we have no way to determine whether or not the participant has sought general practitioner care, a preventer medication has yet been prescribed, the participant has collected the medication from another pharmacy, or the participant has chosen to not have the item dispensed for any personal reasons, including cost, adverse effects, or personal beliefs and knowledge about asthma and its management. However, this is a population (those with uncontrolled asthma and poor adherence) that pharmacists can assist in identifying and initiating care pathways for by referring them to their general practitioner. Greater vigilance regarding patient preventer medication dispensing or lack thereof will assist in minimizing this proportion of at-risk patients. 

There is still a proportion of the participants whose lack of control is not explained by poor adherence. Therefore, more investigation is required to determine why control has not been achieved in this population despite adherence to medications. Other possible explanations include poor inhaler technique, suboptimal prescribing of respiratory medication, discarding medications that have been collected, the presence of severe asthma requiring specialist care, or the presence of untreated or poorly managed co-morbidities that are known to affect asthma control, such as gastro-oesophageal reflux disease [13,54], sleep-related issues such as sleep apnoea [55], obesity [13,56], and depression or anxiety [13,57,58].

One in four study participants were using add-on respiratory agents in addition to the asthma medications specified in international guidelines, including the addition of LAMA, SAMA, LAMA + LABA combinations or triple therapy. This may be indicative of advanced Step 4 therapy or Step 5 therapy—the highest level of asthma management [13,23]. It is difficult to determine if this is in fact an indication of severe asthma, the presence of Asthma–COPD Overlap Syndrome, inappropriate prescribing, a lack of fidelity to the exclusion criteria which asked pharmacists to exclude people with COPD, or the participants’ understanding of their own diagnosis [59]. Studies have reported a lack of recognition by participants of COPD diagnosis, and diagnostic confusion between COPD and asthma [60,61]. Often, if asthma was the lifelong diagnosis, this diagnosis title can remain and patients do not see COPD as a separate diagnosis, or they may not be told they have COPD [62].

A high proportion of participants presented with comorbid allergic rhinitis (71%), which is close to population estimates for those with asthma [13]. Suboptimal management of allergic rhinitis was apparent, as half of the cohort did not report using any treatments to address their symptoms. The optimal therapy for participants with uncontrolled asthma and allergic rhinitis is the regular use of an intranasal corticosteroid. However, just over one in five participants who were asked about their allergic rhinitis management were using an intranasal corticosteroid; this should be higher. Thus, poorly controlled allergic rhinitis may be one of the factors influencing the lack of asthma control in our community cohort, although our exploratory analysis and regression did not show this.

The univariate analysis indicated that those more likely to be adherent were participants who had had a hospitalization, were older or were patients with a health care concession, which is not surprising. A hospitalization for asthma would focus the person with asthma on the need to control their disease with the appropriate use of medication. This could be an opportunity for pharmacists to maintain this awareness and focus on how to keep well. Furthermore, it has been shown that younger adults have lower rates of adherence than older adults with asthma [63,64,65,66,67]. Despite previous studies suggesting other risks associated with poor adherence, the number of medications and the severity of the disease was not associated with better/poorer adherence in our cohort of participants. In Australia, some residents, including seniors, government social security allowance recipients and low-paid workers, are eligible for health care concessions, which allow access to prescription items and medical services at a discounted rate. For some asthma preventer medications, this could mean a saving of AUD 34.40 per supply, as per 2020 patient contribution fees [68,69]. It is known that out-of-pocket expenses can be a driving force in patient decision-making regarding adherence to therapy [21,32]. Our results supported this, showing that participants with a health care concession, who pay significantly less for preventer medications, were more adherent than those without. 

Certain types of devices were found to be significantly associated with improved adherence (Ellipta device users) or with lower adherence (Turbuhaler, if this was the only device the participant was using). It could be speculated that because the Turbuhaler has a higher internal resistance than other dry powder inhalers, greater force is required to inhale the required dose. As greater effort is required, participants not using the device correctly may not have been receiving the therapeutic dose, and therefore would not have received the adequate effect. Additionally, the Turbuhaler device may also be deemed somewhat cumbersome as it requires precise positioning (upright) during activation, and inhalation has no perceivable taste or smell sensation. This may have led to the belief that the medication is not effective, or an overall dissatisfaction with the device and impact adherence. Conversely, the Ellipta is easier to use, and medications formulated in this device have only once-daily dosage requirements, which may indicate why Ellipta users were more adherent. Patient satisfaction with respiratory device type has been previously shown to positively impact adherence and improve therapeutic outcomes [70], however preferences are subjective and variable [67,70]. More research is required to examine the association between respiratory device type and patient adherence, as existing studies have produced contradictory outcomes [67,71,72,73]. 

Our results indicate that the identification of people with asthma who need adherence support and education is difficult based on characteristics alone, and greater vigilance is required in monitoring medication collection for each patient.

### Limitations

Preventer medications in Australia are scheduled as prescription only, and so we have a clear data trail for each of these purchases. The medication usage data presented are representative of what has been recorded for each recipient at the recruiting community pharmacy. We cannot be certain that the participant had not collected other medications elsewhere. To help mitigate this issue, inclusion criterion for the trial were included to ensure that the participant was a regular patient at the pharmacy, and the pharmacists received training to ensure this. This criterion allowed us to see dosage instructions so as to measure adherence accurately. Additionally, we can only report on what participants had chosen to get dispensed, which does not mean other medication was not prescribed and not taken to the pharmacy. 

To the best of our knowledge, the application of PDC calculations has not previously been undertaken to determine adherence to asthma therapy. People with asthma are known to self-titrate their medications in response to their symptoms, and so dosage variability may exist. Thus, there may be different perspectives on what constitutes adherence from an individual perspective versus clinical calculation. 

Short-acting beta 2 agonists in Australia were available without a prescription at the time the study was conducted. However, some people purchase them with a prescription if they are eligible for health benefits through a government health care concession, as a large proportion of the cost becomes subsidized. For this reason, the data we have on short-acting beta 2 agonists are only representative of those with a health care concession card, and are missing for the remaining cohort. 

All participant co-morbidities, other respiratory illnesses or recent experiences with acute illness, and the degree to which these may have impacted medicine use and control or impacted on quality of life, are unknown. However, our inclusion criteria for participation asked pharmacists to exclude people with COPD from the trial. Our study relied on patient self-reporting a COPD diagnosis, as we cannot clinically differentiate between asthma and COPD within a pharmacy setting.

Pharmacies were sampled from areas of differing rurality, which matched the distribution of the Australian population, and thus the cohort is representative of our region. In total, 95 pharmacies recruited on average four participants each. There was no significant difference in recruitment rate between the states in Australia. 

## 5. Conclusions

In Australia, it is estimated that approximately half of the people with asthma have poorly controlled asthma [16]. By exploring medication use for the population most at risk of future exacerbations, i.e., those with uncontrolled asthma, we were able to determine medication-related practices that perpetuate poor control. Poor choices at a patient or primary care level have the power to cause further individual- and community-based burdens clinically, socially and economically. Our results support previous research which has shown that adherence to preventer medication in people with asthma is poor in a large proportion of the population with asthma, particularly those who have poorly controlled asthma. 

Health care system improvements are needed to target practices that compromise patient care and that increase the preventable risk associated with the suboptimal medication management of both asthma and allergic rhinitis. Pharmacists are in an excellent position to identify those with poorer adherence via their dispensing records. More work is required to pinpoint target characteristics that can more efficiently identify individuals with adherence issues, and form the inclusion criteria for future programs. 

## Figures and Tables

**Table 1:** Profile of participants (*n* = 363).

Factor	Values	Total *n* (%)
Pharmacy State	New South Wales	257 (70.8)
	Western Australia	64 (17.6)
	Tasmania	42 (11.6)
Pharmacy Remoteness	High Accessible	241 (66.4)
	Accessible/Moderately accessible, remote, and very remote	122 (33.6)
Age	>55 years of age	195 (53.7)
	≤55 years of age	168 (46.3)
Sex	Females	252 (69.4)
	Males	111 (30.6)
Health Care Concession Status	Yes	153 (42.1)
	No	163 (44.9)
	Unspecified	47 (12.9)
Age of asthma onset	≥16 years of age	201 (55.4)
	<16 years of age	162 (44.6)
Lung Function Test	Never	103 (28.4)
	Greater than 12 months ago	164 (45.2)
	Within last 12 months	96 (26.4)
Presentation to hospital in the past 12 months related to asthma	No	275 (75.8)
	Yes	88 (24.2)
At least 1 hospitalization in the past 12 months related to asthma	No	307 (84.6)
	Yes	56 (15.4)
Active Smoker	No	311 (85.7)
	Yes	52 (14.3)
History of allergic rhinitis	Yes	259 (71.3)
	No	104 (28.7)
ACQ Score	Mean	2.49 (± 0.89 SD)
	Median	2.20
	Q1; Q3 (IQR)	1.80; 3.00 (1.20)

**Table 2:** Medications dispensed over the previous 12 months by drug class (*n* = 363 participants).

Medication Type	Frequency *n* (%)
Respiratory medications dispensed (incl. relievers and preventers)	322 (88.7)
Any preventer medication dispensed (ICS ^1^ with or without LABA ^2^, or LTRA ^3^)	292 (80.4)
SABA Reliever ^4^	203 (55.9)
Oral corticosteroid dispensed (Prednisone/Prednisolone 25 mg)	88 (24.2)
ICS + LABA	275 (75.8)
ICS	27 (7.4)
LTRA	6 (1.7)
Theophylline	5 (1.4)
LABA	4 (1.1)
Cromones (Mast Cell stabilizers)	3 (0.8)
Monoclonal Antibody	1 (0.3)
Nebules	34 (9.4)
Salbutamol	30 (8.3)
Ipratropium	14 (3.9)
Add-on therapy ^5^	88 (24.2)
LAMA	68 (18.7)
SAMA	20 (5.5)
LAMA + LABA	11 (3.0)
LABA + LAMA + ICS	8 (2.2)

Note: ^1^ ICS = inhaled corticosteroid; ^2^ LABA = long-acting beta2-agonist; ^3^ LTRA = leukotriene receptor antagonist; ^4^ SABA = short-acting beta2-agonist, SABA recorded does not include that used via nebule; ^5^ Add-on therapy includes LAMA = long-acting muscarinic antagonist, SAMA = short-acting muscarinic antagonists and LAMA + LABA, LABA + LAMA + ICS combinations.

**Table 3:** Medications dispensed by formulation as available in Australia—excluding short-acting reliever medications (*n* = 302 participants).

Device Type	Frequency *n* (%)
Pressurized Metered Dose Inhaler (pMDI)	132 (43.7)
Turbuhaler	75 (24.8)
Accuhaler	73 (24.2)
Ellipta	52 (17.2)
Respimat	33 (10.9)
Handihaler	30 (9.9)
Rapihaler	26 (8.6)
Spiromax	15 (5.0)
Oral tablet	10 (3.3)
Breezehaler	7 (2.3)
Genuair	4 (1.3)
Autohaler	1 (0.3)
Syringe	1 (0.3)
Syrup	1 (0.3)

**Table 4:** Inhaled corticosteroid doses currently taken by participants (*n* = 240 participants).

Medication Class	ICS Strength	Frequency *n* (%)
ICS ^1^ + LABA ^2^		225 (93.8)
	Low ^3^	20 (8.9)
	Medium ^4^	103 (45.8)
	High ^5^	102 (45.3)
ICS ^1^		23 (9.6)
	Low ^3^	3 (13.0)
	Medium ^4^	12 (52.2)
	High ^5^	8 (34.8)

Note: ^1^ ICS = inhaled corticosteroid; ^2^ LABA = long-acting beta2-agonist; ^3^ Low ICS = Beclometasone Dipropionate (100–200 mcg), Budesonide (200–400 mcg), Ciclesonide (80–160 mcg), fluticasone propionate (100–200 mcg); ^4^ Medium ICS = Beclometasone Dipropionate (250–400 mcg), Budesonide (500–800 mcg), Ciclesonide (240–320 mcg), fluticasone furoate (100 mcg), fluticasone propionate (250–500 mcg); ^5^ High ICS = Beclometasone Dipropionate (>400 mcg), Budesonide (>800 mcg), Ciclesonide (>320 mcg), fluticasone furoate (200 mcg), fluticasone propionate (>500 mcg).

**Table 5:** Medications used by those treating allergic rhinitis symptoms (*n* = 152 participants).

Participants Treating Allergic Rhinitis Symptoms	Frequency *n* (%)
Yes	77 (49.3)
No	75 (50.7)
**Medication Combinations Used**	
Oral antihistamine	31 (20.4)
Oral antihistamine + intranasal corticosteroid	14 (9.2)
Intranasal corticosteroid	9 (5.9)
Oral antihistamine + intranasal corticosteroid + intranasal saline	4 (2.6)
Oral antihistamine + intranasal decongestant	4 (2.6)
Oral antihistamine + oral decongestant	3 (2.0)
Oral antihistamine + intranasal corticosteroid + ocular antihistamine	2 (1.3)
Oral antihistamine + intranasal saline	2 (1.3)
Oral antihistamine + intranasal antihistamine	1 (0.7)
Oral antihistamine + ocular antihistamine	1 (0.7)
Oral antihistamine + ocular antihistamine + oral decongestant	1 (0.7)
Oral antihistamine + intranasal corticosteroid + intranasal saline + ocular saline	1 (0.7)
Oral antihistamine + intranasal decongestant + intranasal corticosteroid + intranasal saline	1 (0.7)
Intranasal corticosteroid + intranasal saline	1 (0.7)
Intranasal corticosteroid + oral decongestant	1 (0.7)
Ocular antihistamine	1 (0.7)

## Abbreviations

ACQ	Asthma Control Questionnaire
COPD	Chronic Obstructive Pulmonary Disease
GINA	Global Initiative for Asthma
ICS	Inhaled corticosteroid
LTRA	Leukotriene receptor antagonist
LABA	Long-acting beta2-agonist
LAMA	Long-acting muscarinic antagonist
NAC	National Asthma Council Australia
pMDI	Pressurized Metered Dose Inhaler
PDC	Proportion of Days Covered
SABA	Short-acting beta2-agonist
SAMA	Short-acting muscarinic antagonists

## Appendix A. Medication Dispensed by Brand Name and Strength

Asthma preventative medications dispensed in the 12 months prior to data collection by formulation, trade name and strength are presented in Table A1.

**Table A1:** Asthma preventers dispensed over the previous 12 months (*n* = 292 participants).

Formulation	Trade Name	Strength	Frequency *n* (%)
Fluticasone Propionate/Salmeterol	Seretide MDI	250 mcg/25 mcg	85 (29.1)
Budesonide/Formoterol Fumarate Dihydrate	Symbicort Turbuhaler	200 mcg/6 mcg	42 (14.4)
Fluticasone Propionate/Salmeterol	Seretide Accuhaler	250 mcg/50 mcg	36 (12.3)
Fluticasone Propionate/Salmeterol	Seretide Accuhaler	500 mcg/50 mcg	27 (9.2)
Budesonide/Formoterol Fumarate Dihydrate	Symbicort Turbuhaler	400 mcg/12 mcg	27 (9.2)
Budesonide/Formoterol Fumarate Dihydrate	Symbicort Rapihaler	200 mcg/6 mcg	24 (8.2)
Fluticasone Furoate/Vilanterol	Breo Ellipta	200 mcg/25 mcg	18 (2.2)
Fluticasone Furoate/Vilanterol	Breo Ellipta	125 mcg/25 mcg	15 (6.2)
Budesonide/Formoterol Fumarate Dihydrate	DuoResp Spiromax	400 mcg/12 mcg	12 (4.1)
Ciclesonide	Alvesco MDI	160 mcg	11 (3.8)
Fluticasone Propionate	Flixotide, Fluticasone Cipla MDI	250 mcg	8 (2.7)
Fluticasone Propionate/Salmeterol	Seretide, F/S Cipla, Salplusf MDI	125 mcg/25 mcg	8 (2.7)
Fluticasone Propionate	Flixotide, Fluticasone Cipla MDI	125 mcg	7 (2.4)
Montelukast	Singulair, Respikast, Lukaira, Montelukast Tabs	10 mg	6 (2.1)
Fluticasone Propionate	Flixotide Accuhaler	250 mcg	5 (1.7)
Fluticasone Propionate/Formoterol Fumarate Dihydrate	Flutiform MDI	250 mcg/10 mcg	5 (1.7)
Fluticasone Propionate/Formoterol Fumarate Dihydrate	Flutiform MDI	125 mcg/5 mcg	4 (1.4)
Theophylline	Nuelin SR	250 mg	4 (1.4)
Budesonide	Pulmicort Turbuhaler	400 mcg	3 (1.0)
Budesonide/Formoterol Fumarate Dihydrate	DuoResp Spiromax	200 mcg/6 mcg	3 (1.0)
Salmeterol	Serevent Accuhaler	50 mcg	2 (0.7)
Fluticasone Propionate/Salmeterol	Seretide Accuhaler	100 mcg/50 mcg	2 (0.7)
Budesonide/Formoterol Fumarate Dihydrate	Symbicort Turbuhaler	100 mcg/6 mcg	2 (0.7)
Budesonide/Formoterol Fumarate Dihydrate	Symbicort Rapihaler	100 mcg/3 mcg	2 (0.7)
Formoterol Fumarate Dihydrate	Oxis Turbuhaler	6 mg	1 (0.3)
Sodium Cromoglycate	Intal MDI	1 mg	1 (0.3)
Sodium Cromoglycate	Intal MDI	5 mg	1 (0.3)
Nedocromil Sodium	Tilade MDI	2 mg	1 (0.3)
Fluticasone Propionate	Flixotide Accuhaler	500 mcg	1 (0.3)
Beclometasone Dipropionate	Qvar Autohaler	100 mcg	1 (0.3)
Fluticasone Propionate/Salmeterol	Seretide, F/S Cipla, Salplusf MDI	50 mcg/25 mcg	1 (0.3)
Omalizumab	Xolair Syringe		1 (0.3)
Theophylline	Nuelin Syrup	133.3 mg/25 mL	1 (0.3)

## Appendix B. Combination Therapy

Combinations of medications dispensed in the 3 months prior to data collection are presented in Table A2, Table A3 and Table A4.

**Table A2:** Combination therapy in 3 months preceding control assessment—All respiratory medications (*n* = 280 participants).

Medication Combination	Frequency *n* (%)
(ICS + LABA) (SABA)	88 (31.4)
(ICS + LABA)	77 (27.5)
(SABA)	25 (8.9)
(ICS + LABA) (SABA)(LAMA)	22 (7.9)
(ICS + LABA) (LAMA)	14 (5.0)
(ICS)	6 (2.1)
(ICS + LABA) (Cromone)	4 (1.4)
(ICS) (ICS + LABA) (LAMA)	3 (1.1)
(ICS + LABA) (SABA) (SAMA)	3 (1.1)
(LAMA)	3 (1.1)
(SABA) (LAMA)	3 (1.1)
(ICS) (ICS + LABA) (SABA)	2 (0.7)
(ICS) (LABA)	2 (0.7)
(ICS) (SABA)	2 (0.7)
(ICS) (SABA) (LAMA + LABA)	2 (0.7)
(LAMA + LABA + ICS)	2 (0.7)
(SABA) (LAMA + LABA)	2 (0.7)
(SABA) (LAMA + LABA + ICS)	2 (0.7)
(ICS) (ICS + LABA)	1 (0.4)
(ICS) (ICS + LABA) (SABA) (LAMA)	1 (0.4)
(ICS) (ICS + LABA) (SABA) (LAMA) (LAMA + LABA)	1 (0.4)
(ICS) (LABA) (SABA) (LAMA)	1 (0.4)
(ICS) (LAMA + LABA)	1 (0.4)
(ICS) (SABA) (THEOPH)	1 (0.4)
(ICS + LABA) (LAMA) (LAMA + LABA)	1 (0.4)
(ICS + LABA) (LTRA)	1 (0.4)
(ICS + LABA) (MONOAB)	1 (0.4)
(ICS + LABA) (Cromone) (LAMA + LABA)	1 (0.4)
(ICS + LABA) (Cromone) (SABA)	1 (0.4)
(ICS + LABA) (Cromone) (SABA) (LAMA)	1 (0.4)
(ICS + LABA) (SABA) (LAMA)(LAMA + LABA + ICS)	1 (0.4)
(ICS + LABA) (SABA) (LAMA) (SAMA)	1 (0.4)
(ICS + LABA) (THEOPH) (LAMA)	1 (0.4)
(LAMA + LABA)	1 (0.4)
(LTRA) (SABA) (LAMA + LABA + ICS)	1 (0.4)
(SABA) (SAMA) (LAMA + LABA + ICS)	1 (0.4)

**Table A3:** Combination therapy in 3 months preceding control assessment—Asthma medications only (*n* = 274 participants).

Medication Combination	Frequency *n* (%)
(ICS + LABA) (SABA)	115 (42.0)
(ICS + LABA)	92 (33.6)
(SABA)	33 (12.0)
(ICS)	7 (2.6)
(ICS + LABA) (Cromone)	5 (1.8)
(ICS) (ICS + LABA)	4 (1.5)
(ICS) (ICS + LABA) (SABA)	4 (1.5)
(ICS) (SABA)	4 (1.5)
(ICS) (LABA)	2 (0.7)
(ICS + LABA) (Cromone) (SABA)	2 (0.7)
(ICS) (LABA) (SABA)	1 (0.4)
(ICS) (SABA) (THEOPH)	1 (0.4)
(ICS + LABA) (LTRA)	1 (0.4)
(ICS + LABA) (MONOAB)	1 (0.4)
(ICS + LABA) (THEOPH)	1 (0.4)
(LTRA) (SABA)	1 (0.4)

**Table A4:** Combination therapy in 3 months preceding control assessment—Add-on therapies (*n* = 69 participants).

Medication Combination	Frequency *n* (%)
(LAMA)	49 (71.0)
(LAMA + LABA)	7 (10.1)
(LAMA + LABA + ICS)	5 (7.2)
(SAMA)	3 (4.3)
(LAMA) (LAMA + LABA)	2 (2.9)
(LAMA) (LAMA + LABA + ICS)	1 (1.4)
(LAMA) (SAMA)	1 (1.4)
(SAMA) (LAMA + LABA + ICS)	1 (1.4)
